# Cytotoxicity profiling of deep eutectic solvents to human skin cells

**DOI:** 10.1038/s41598-019-39910-y

**Published:** 2019-03-08

**Authors:** I. P. E. Macário, H. Oliveira, A. C. Menezes, S. P. M. Ventura, J. L. Pereira, A. M. M. Gonçalves, J. A. P. Coutinho, F. J. M. Gonçalves

**Affiliations:** 1 Department of Biology & CESAM, University of Aveiro, 3810-193 Aveiro, Portugal; 2 CICECO - Aveiro Institute of Materials & Department of Chemistry, University of Aveiro, 3810-193 Aveiro, Portugal; 3 MARE, Department of Life Sciences, Faculty of Sciences and Technology, University of Coimbra, 3004-517 Coimbra, Portugal

## Abstract

The tailor-made character of deep eutectic solvents (DES) turns them very attractive to be used in several applications, including in health-related areas such as pharmaceutical, nutraceutical, and cosmetic industries. However, although DES has been touted as “green” solvents, several works proved that their potential toxicity should not be neglected. Using the premise of DES applicability in the cosmetic and pharmaceutical sectors, we chose two cell lines to work as a skin model (keratinocytes HaCaT and tumor melanocytes MNT-1), to assess DES cytotoxicity. The effect of three different hydrogen bond acceptors (HBA) ([Chol]Cl, [N_1111_]Cl and [N_4444_]Cl) and three different hydrogen bond donors (HBD) (hexanoic and butanoic acid, ethylene glycol, 1-propanol and urea) were evaluated through a common viability assay (MTT assay). Results were promising since [Chol]Cl and [N_1111_]Cl- based DES showed good biocompatibility for the tested cells. [N_4444_]Cl-based DES, however, showed cytotoxicity for both cell lines, with the HBA being the driver of the toxicity. Interestingly, some compounds increased cell viability in the HaCaT cell line, namely [Chol]Cl, ethylene glycol, hexanoic acid, urea, and all [Chol]Cl and [N_1111_]Cl-based DES and should be considered as targets for future studies. These results highlight their possible use in cosmetic or pharmaceutical formulations.

## Introduction

The field of “designer solvents” such as ionic liquids (IL) and deep eutectic solvents (DES) has been growing in the past decades, under the scope of “Green Chemistry”, which promotes the design and application of chemical products and processes that could reduce or preferentially eliminate the use and generation of hazardous substances^1^. DES were firstly developed in 2003 by combining urea and cholinium chloride^2^. These are prepared through the mixing of two or three different starting materials (e.g., quaternary ammonium salts, amides, organic acids, polyalcohols) forming an eutectic mixture based on hydrogen bonding interactions between a hydrogen bond donor (HBD) and an acceptor (HBA). These present a melting point much lower than either of the individual components^2–5^. This significant decrease in the melting point compared to starting materials is the result of several factors, such as the interaction between the salt’s anionic species and the HBD, the lattice energies, the nature and asymmetry of the organic salts, and the charges delocalization through the hydrogen bonding^4^. These new solvents are simpler to prepare and do not need complex purification schemes^3,4^. Moreover, DES are recognized as having a cheap production, (due to the low cost of starting materials), and showing a good biocompatibility with different biomolecules^6–8^.

The possible aplications for DES are almost endless owing to their designer character, and presently are mainly focused on chemical, electrochemical and material applications^9^. More recently, health-related industries such as the pharmaceutical, nutraceutical and cosmetic are also exploring these compounds due to their compatibility with biomolecules like DNA and enzymes^10^, among others. DES are suitable for biotransformation processes^11^, as well as to process biomass^12,13^, perform extractions^14^ and stabilize natural pigments^15^ as reviewed by Mbous *et al*.^16^. DES based on natural compounds, such as primary metabolites, like organic acids, amino acids and sugars^5,10^ have been labelled Natural Deep Eutectic Solvents (NADES). Nowadays, their study is a promising area in the field of cellular metabolism and physiology. Some authors (Choi *et al*.^10^) consider that these solvents could be involved on the biosynthesis of non-water soluble molecules and can act as solvents in living organisms, as water and lipids. DES may be particularly interesting for cosmetic proposes^17,18^, since the extraction technology using DES can mimic the processes that plants use to solubilize their essential molecules (flavonoids, anthocyanins and polymers). Moreover, the DES physical and chemical properties render them highly efficient in solubilizing compounds that are normally poorly water- or lipid-soluble^17^. In addition, social awareness turns consumers increasingly interested in low-toxic and natural solvents^17^. Although DES were initially considered as “green solvents”, mainly due to the benign nature of their constituents, only a few studies are available that assessed their toxic potential, either regarding their ecotoxicity^6,19–27^, or their cytotoxicity^22,23,28–30^. These few studies show that the DES toxic profile should be better characterized before general classifications of their benign character can be assumed. Moreover, information about the toxicity of DES is critical for a proper risk assessment under regulatory frameworks worldwide (e.g. the REACH regulation in Europe, which ensure the safety of chemical products for people and environment^31^).

This work aims to assess the cytotoxicity of a set of DES towards two human skin cell lines, HaCaT (keratinocytes cells) and MNT-1 (melanoma cells), considering the increased relevance of these solvents in many sectors of industry. HaCaT^32–35^, in particular, was chosen as model for cosmetic applications, while MNT-1^36–38^ was selected as a model to understand the potential of the DES under study on the treatment of skin disorders. The DES effects on the cell viability were assessed through the MTT assay. More specifically, the effects of three different HBA and three different structural groups as HBD in DES cytotoxicity were evaluated supporting the drawing of informative toxicity trends. In this way, we tested fifteen DES at a 1:1 molar ratio (HBA:HBD), and rationally selected the starting materials. Two ammonium chlorides with different alkyl chain lengths (the larger the most toxic was the underlying assumption^39^), and cholinium chloride, which has been argued as biocompatible^40,41^, were selected as HBA. Then, two alcohols and two acids differing in their alkyl chain length, and number of functional groups, as well as an amine (urea) were selected to represent the most commonly functional groups applied as HBD for DES formulation.

## Materials and Methods

### Cell culture

Human melanoma MNT-1 cells were generously provided by Doctor Manuela Gaspar (iMed.ULisboa, Portugal). Immortalized human keratinocyte HaCaT cells were obtained from Cell Lines Services (Eppelheim, Germany). MNT-1 and HaCaT cells were cultured in Dulbecco’s modified Eagle’s medium (DMEM) supplemented with 10% of fetal bovine serum (FBS) and 1% of L-glutamine, penicillin–streptomycin and fungizone (Life Technologies, Grand Island, NY, USA). Both cell cultures were incubated in a humidified atmosphere at 37 °C and 5% of carbon dioxide – CO_2_. Cell morphology was observed using an inverted microscope Nikon Eclipse 80i (Nikon, Tokyo, Japan).

### DES preparation

The following chemical compounds were used for DES preparation. As HBA, cholinium chloride ([Chol]Cl − 98% of purity) was purchased from Acros Organic^®^; tetramethylammonium chloride ([N_1111_]Cl − 97% of purity) and tetrabutylammonium chloride [N_4444_]Cl − 97% of purity) were purchased from Sigma-Aldrich. As HBD, ethylene glycol (99.5% of purity) was purchased from Sigma-Aldrich; 1-propanol (99.5% of purity) was purchased from Merck; butanoic acid (99% of purity) was purchased from Riedel de Haën; hexanoic acid (98% of purity) was purchased from SAFC; and urea (99% of purity) from Panreac. All DES were prepared at a molar ratio of 1:1, HBA:HBD. Briefly, HBDs and HBAs were added gravimetrically to closed vials and heated in a heat block with constant agitation. After the formation of a transparent liquid, the mixture was cooled down to room temperature. For some of these eutectic mixtures a known volume of water was added. The water content of both starting materials and DES was determined by Karl Fischer titration as detailed elsewhere^42^ and considered in calculations regarding cytotoxicity benchmarks.

### MTT assay

The cytotoxic effects of HBAs, HBDs and DES were assessed by the colorimetric MTT assay^43^. Briefly, MNT-1 and HaCaT cells were seeded in 96-well plates and allowed to adhere. After adhesion, cells were incubated for 72 h exposed to a range of six concentrations (50–500 µg.mL^−1^), of the tested compounds diluted in DMEM medium (these previously sterilized with a 0.22 µm syringe filter), at 37 °C in 5% of CO_2_. After 72 h of exposure, 50 µL of MTT solution (3-(4,5-dimethylthiazol-2-yl)-2,5-diphenyltetrazolium bromide (MTT) from Sigma-Aldrich) (1 mg.mL^−1^ in PBS, pH 7.2) was added to each well. After 4 h of incubation, the medium was replaced with 150 µL of dimethyl sulfoxide (DMSO) to dissolve the formazan crystals. The plate was shaken for approximately 2 h, protected from light. Cell viability was measured through the optical density of reduced MTT at 570 nm using a microplate reader (Synergy HT from BioTeK Instruments Inc., Winooski, VT, USA). The percentage of viable cells was calculated as the ratio between the absorbance of treated *versus* control cells. Likewise, IC_50_ was defined as the concentration of chemical that leads to a 50% decrease in cell viability, calculated through a non-linear regression, logistic function.

### Statistical analysis

Data were expressed as the mean ± standard deviation (SD) of at least three independent experiments with three technical replicates each. Data from each test (with HBD, HBA and DES) were analysed by one-way ANOVA, followed by a Dunnett’s test to evaluate the significance of disparities between the treatment groups and the control. In the absence of normality or homogeneity of variances, as assessed with Shapiro-Wilk and Brown-Forsythe tests, respectively, data were analysed by non-parametric one-way ANOVA (Kruskall-Wallis) followed by Dunnett’s test (only for [N_1111_]Cl:1-propanol in HaCaT cell line, the Dunn’s tests was used instead, due to unequal samples size). A value of *p* < 0.05 was considered statistically significant.

## Results

In this study, keratinocyte cells (HaCaT) were used as a model of non-tumoral skin cells, and melanoma cells (MNT-1 cells) were used as a model of skin tumor cells. Most of the compounds showed to be non-cytotoxic after 24 and 48 h of exposure at low concentrations in preliminary trials. Thus, the concentration range was increased (0–500 µg.mL^−1^), as well as the exposure time for 72 h, in order to discard the possibility of toxic effects under more dramatic conditions.

### Cytotoxicity of HBA and HBD

From the HBAs under study, only [N_4444_]Cl was found to be toxic for both cell types after 72 h of single exposure (see Table 1 and Supplementary Figs S1, S3). Regarding the HBDs, only butanoic acid showed a toxic effect for both cell types. [N_1111_]Cl did not exert any effect in cell viability in MNT-1, but increased the viability of HaCaT at 50 μg.mL^−1^ and significantly decreased it at 500 μg.mL^−1^. On the opposite, 1-propanol also did not exert any effect in cell viability in HaCaT, but significantly increased the viability in MNT-1 cells following exposure to all tested concentrations. Hexanoic acid increased cell viability in HaCaT, but produced a slight, yet statistically significant, reduction considering the higher concentrations towards the MNT-1 cells. All the remaining compounds increased cell viability at both cell lines (see Table 1 and Supplementary Figs S1, S3). A least pronounced effect was caused by [Chol]Cl, which only produced a significant increase at the concentration 50, 200 and 300 μg.mL^−1^ for the HaCaT cell line, and at the concentration of 200 μg.mL^−1^ for MNT-1 cells. Ethylene glycol caused the higher increase in cell viability especially in HaCaT cells, with some treatments reaching 40% of increase in cell viability compared to the control (see Supplementary Figs S1, S3).

**Table 1:** Summary of the effects of HBAs and HBDs, on the two studied cell lines, after 72 h of exposure.

	Chemical compounds	Cell line	Effect trend	IC_50_ ± SD (μg.mL^−1^)	LOEC (μg.mL^−1^)
HBA	[Chol]Cl	HaCaT MNT-1	Increase (slightly) Increase (slightly)	— —	50 200
	[N_1111_]Cl	HaCaT MNT-1	No effect No effect	— —	50 —
	[N_4444_]Cl	HaCaT MNT-1	Toxic effect Toxic effect	220.8 ± 6.2 316.1 ± 25.7	200 50
HBD	Butanoic acid	HaCaT MNT-1	Toxic effect Toxic effect	309.2 ± 11.6 274.8 ± 5.0	— —
	Hexanoic acid	HaCaT MNT-1	Increase Toxic (slightly)	— —	50 50
	Ethylene glycol	HaCaT MNT-1	Increase Increase	— —	50 50
	1-Propanol	HaCaT MNT-1	No effect Increase	— —	— 50
	Urea	HaCaT MNT-1	Increase Increase	— —	100 50

The effect trend concerns the effect of the chemicals in cell viability. Inhibitory concentration values (IC_50_) and Lowest Observed Effect Concentration (LOEC) (Dunnett test following one-way ANOVA; p < 0.05; see Supplementary Table S1 for the ANOVA summary) are also presented.

### Cytotoxicity of DES

Cytotoxicity of DES is summarised in Table 2 and depicted in Supplementary Figs S2, S4. All [Chol]Cl-based DES promoted an increase in cell viability of HaCaT cells. The highest increase was found following exposure to [Chol]Cl:butanoic acid, reaching almost 40% at 50 and 100 µg.mL^−1^; however at 500 µg.mL^−1^, a decrease in cell viability was observed. This DES produced interesting results, since it was the only one promoting the cell viability in HaCaT, but it showed a toxic effect in MNT-1, at higher concentrations. However, and although significant, the decrease in MNT-1 cell viability was measured only up to 20% at the highest concentration tested (500 µg.mL^−1^), which prevented the calculation of median inhibitory concentrations. In MNT-1 cells, the viability increase observed following exposure to [Chol]Cl:ethylene glycol and [Chol]Cl:1-propanol (significant at 50, 100, 300 and 400 µg.mL^−1^) was smaller compared to the viability increase observed in HaCaT. Also for MNT-1 cells, [Chol]Cl:hexanoic acid and [Chol]Cl:urea produced no significant effects regarding cell viability.

**Table 2:** Summary of the effect of the tested DES, for the two studied cell lines, after 72 h of exposure. The effect trend concerns the effect of the chemicals in cell viability.

	HBA: HBD 1:1	Cell line	Effect trend	IC_50 ± _SD (μg.mL^−1^)	LOEC (μg.mL^−1^)
DES	[Chol]Cl: Butanoic acid	HaCaT MNT-1	Increase Toxic (slightly)	— —	50 400
	[Chol]Cl: Hexanoic acid	HaCaT MNT-1	Increase No effect	— —	100 —
	[Chol]Cl: Ethylene glycol	HaCaT MNT-1	Increase Increase	— —	50 50
	[Chol]Cl: 1-Propanol	HaCaT MNT-1	Increase Increase	— —	50 50
	[Chol]Cl: Urea	HaCaT MNT-1	Increase No effect	— —	50 —
	[N_1111_]Cl: Butanoic acid	HaCaT MNT-1	Increase Toxic (slightly)	— —	50 500
	[N_1111_Cl: Hexanoic acid	HaCaT MNT-1	Increase Increase	— —	50 50
	[N_1111_]Cl: Ethylene glycol	HaCaT MNT-1	Increase Increase	— —	50 50
	[N_1111_]Cl: 1-Propanol	HaCaT MNT-1	Increase Increase	— —	50 50
	[N_1111_]Cl: Urea	HaCaT MNT-1	Increase Increase (slightly)	— —	50 50
	[N_4444_]Cl: Butanoic acid	HaCaT MNT-1	Toxic effect Toxic effect	108.7 ± 4.2 472.6 ± 21.3	200 50
	[N_4444_]Cl: Hexanoic acid	HaCaT MNT-1	Toxic effect Toxic effect	112.0 ± 4.3 476.1 ± 23.2	200 100
	[N_4444_]Cl: Ethylene glycol	HaCaT MNT-1	Toxic effect Toxic effect	34.1 ± 5.0 300.9 ± 12.7	200 200
	[N_4444_]Cl: 1-Propanol	HaCaT MNT-1	Toxic effect Toxic effect	77.9 ± 6.3 250.5 ± 7.6	200 50
	[N_4444_]Cl: Urea	HaCaT MNT-1	Toxic effect Toxic effect	81.8 ± 3.1 496.7 ± 58.4	50 200

Inhibitory concentration values (IC_50_) and Lowest Observed Effect Concentration (LOEC) (Dunnett test following one-way ANOVA; p < 0.05; see Supplementary Tables S1 and S2 for the ANOVA summary) is also presented.

[N_1111_]Cl-based DES showed almost no toxicity for HaCaT and for MNT-1 cells. There was a slight cytotoxic effect observed following exposure to [N_1111_]Cl:butanoic acid towards both cell lines, but only at the highest concentration tested (500 µg.mL^−1^), while at the remaining concentrations an increase in cell viability was observed for HaCaT. Still regarding HaCaT, all [N_1111_]Cl-based DES produced an increase in cell viability, which is higher in [N_1111_]Cl:hexanoic acid by reaching the 40%. [N_1111_]Cl:hexanoic acid, [N_1111_]Cl:1-propanol and [N_1111_]Cl:ethylene glycol also increased MNT-1 viability in some treatments. Similarly to [Chol]Cl:urea, [N_1111_]Cl:urea induced no effects in MNT-1 cell viability except for the 50 µg.mL^−1^, where a significant increase of 20% was noticed.

**Figure 1:**
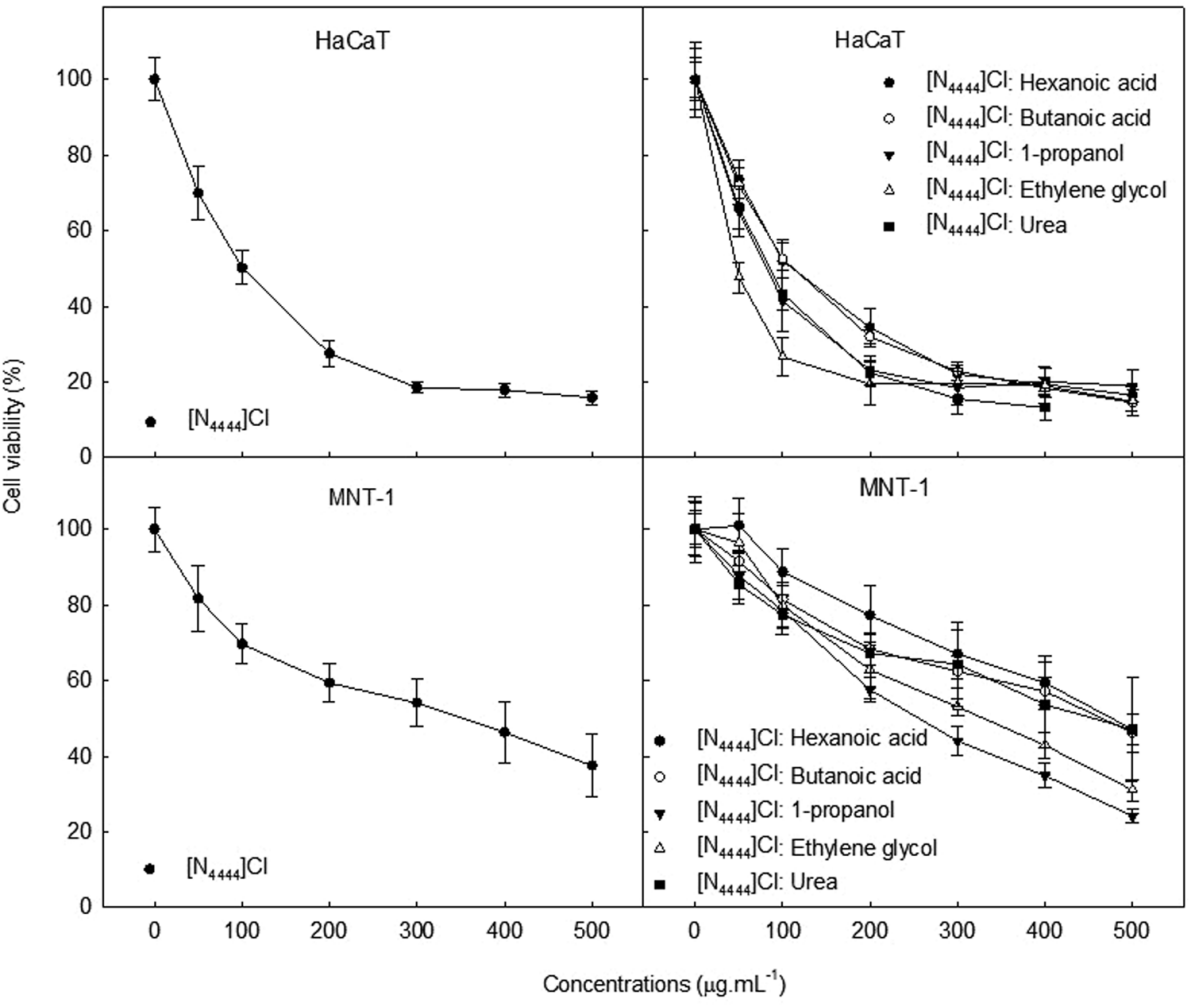
Cell viability of [N_4444_]Cl (starting material) and [N_4444_]Cl-based DES for HaCaT and MNT-1 cells, after 72 h of exposure. Results are expressed as mean ± SD of three independent experiments.

Considering [N_4444_]Cl-based DES, these bearing larger alkyl chains in comparison with the [N_1111_]Cl-based DES, all showed toxicity for both cell lines, regardless the HBD used. Such as [N_4444_]Cl, they were more toxic to HaCaT than to MNT-1 (compare IC_50_ values in Table 2). The most toxic DES with [N_4444_]Cl as HBA was [N_4444_]Cl:ethylene glycol, with an IC_50_ = 34.1 µg.mL^−1^ estimated for HaCaT cells. It is worth further noticing that the toxicity profile of [N_4444_]Cl-based DES is very similar to that observed for the starting material [N_4444_]Cl (see Fig. 1).

## Discussion

The study of DES toxicity is still in its infancy. Regarding their (eco)toxicity, the best studied systems are [Chol]Cl and phosphonium-based DES, which were assessed through a variety of organisms such as bacteria (*Bacillus subtilis*, *Staphylococcus aureus*, *Pseudomonas aeruginosa*^6,19^, *Escherichia coli*^6,19,20^, *Aliivibrio fischeri*^21^, *Listeria monocytogenes, Salmonella enteriditis*^24^); fungi (*Phanerochaete chrysosporium, Candida cylindracea, Aspergillus niger, Lentinus tigrinus*^25^, *Sacharomyces cerevisae*^26^); wheat seeds (*Triticum aestivum*)^22^; invertebrates, (*Hydra sinensis*^20,27^ and *Artemia salina*^6,19^); the plant *Allium sativum*^20^; the fish *Cyprinus carpio*^25^; and mice^23^.

The cytotoxicity of DES was also briefly addressed using different cell lines, *vis*. L929 fibroblast-like cells^28^, PC3 human prostate cancer, A375 human malignant melanoma^23,29^, OKF6 human oral keratinocyte, H413 carcinoma-derived human oral keratinocyte^23^, MCF-7 human breast cancer^22,23,29,30^, HelaS3 human cervical cancer^29,30^, CaOV3 human ovarian cancer, B16F10 mouse skin cancer^30^, AGS human gastric cancer, WRL-68 human hepatocyte^29^, and also CCO fish cells towards a non-human perspective^22^. While Hayyan *et al*.^23^ found that the studied [Chol]Cl-based DES exert relatively high cytotoxicity towards all cell lines, argued as higher than that by their individual components (*i.e*. glycerine, ethylene glycol, triethylene glycol and urea), Radošević *et al*.^22^ found that some DES (*i.e*.[Chol]Cl:oxalic acid) exerted moderate toxicity, while others (*i.e*.[Chol]Cl:glucose and [Chol]Cl:glycose) showed very low cytotoxicity (>2000 mg.L^−1^). Hayyan *et al*.^30^ and Paiva *et al*.^28^ found that NADES prepared with organic acids as HBD (*e.g*. malonic acid, citric acid and tartaric acid) were more cytotoxic to HelaS3, CaOV3, MCF-7, B16F10, and L929 cells. Through the main results, Hayyan *et al*.^23^ admitted that the HBD played a significant role in cytotoxicity. Finally, Mbous *et al*.^29^ used two [Chol]Cl-based NADES and a different DES (N,N-diethyl ethanolammonium chloride:triethylene glycol), and found that although all affect the viability of the tested cell lines, the DES was more toxic than the tested NADES.

In the present study, the cell models used reflected the potential applications of DES in the cosmetics and skin care industry. HaCaT^32–35^ and MNT-1^36–38^ cell lines are widely used as human skin cell models. Surprisingly, given the previous records found in literature (see above), the tested DES did not show cytotoxicity with the exception of all [N_4444_]Cl-based DES, and many were able to stimulate cell viability instead, especially in non-tumoral cells. This shows that they could be considered as promising candidates for use in the cosmetic and/or pharmaceutical sectors.

Not much is known regarding the mechanisms of toxic action of DES and their starting materials, although the interaction with the biological membranes, as well as membrane damage possibly linked with oxidative stress imbalance, that cannot be hold by the antioxidant defence, have been argued relevant in this context^23,29^. Biological membranes are composed by a matrix of lipids and proteins, which regulates their permeability. One of the most important constituents of the lipid bilayer are phospholipids^44^, whose distribution across the membrane creates a membrane potential that regulates permeability and the diffusion of ionic and molecular species. Phospholipids consist on a ratio of functional groups on the cell surface (carboxyl, phosphate and amino groups), which depends on the cell type. The ratio between these functional groups determines the entry and the entry rate of extracellular materials^44^. According to Hayyan *et al*.^23^ and Mbous *et al*.^29^, the cytotoxic mechanism of DES involves an increase in the membrane permeability. Then, once inside the cell, the DES contributes to the increase in the concentration of reactive oxygen species (ROS), challenging the oxidative status of the cell. This could be the case of butanoic acid, [N_4444_]Cl and [N_4444_]Cl-based DES. Indeed, all of these compounds harm both non-tumoral (HaCaT) and tumor cells (MNT-1), probably through a similar mechanism. This was already observed by Mbous *et al*.^29^ with [Chol]Cl:fructose, [Chol]Cl:glucose and N,N-diethylethanolammonium chloride:triethylene glycol, in cancer cell lines (HelaS3, PC3, AGS, A375 and MCF-7) and the non-tumoral cell line WRL-68.

**Figure 2:**
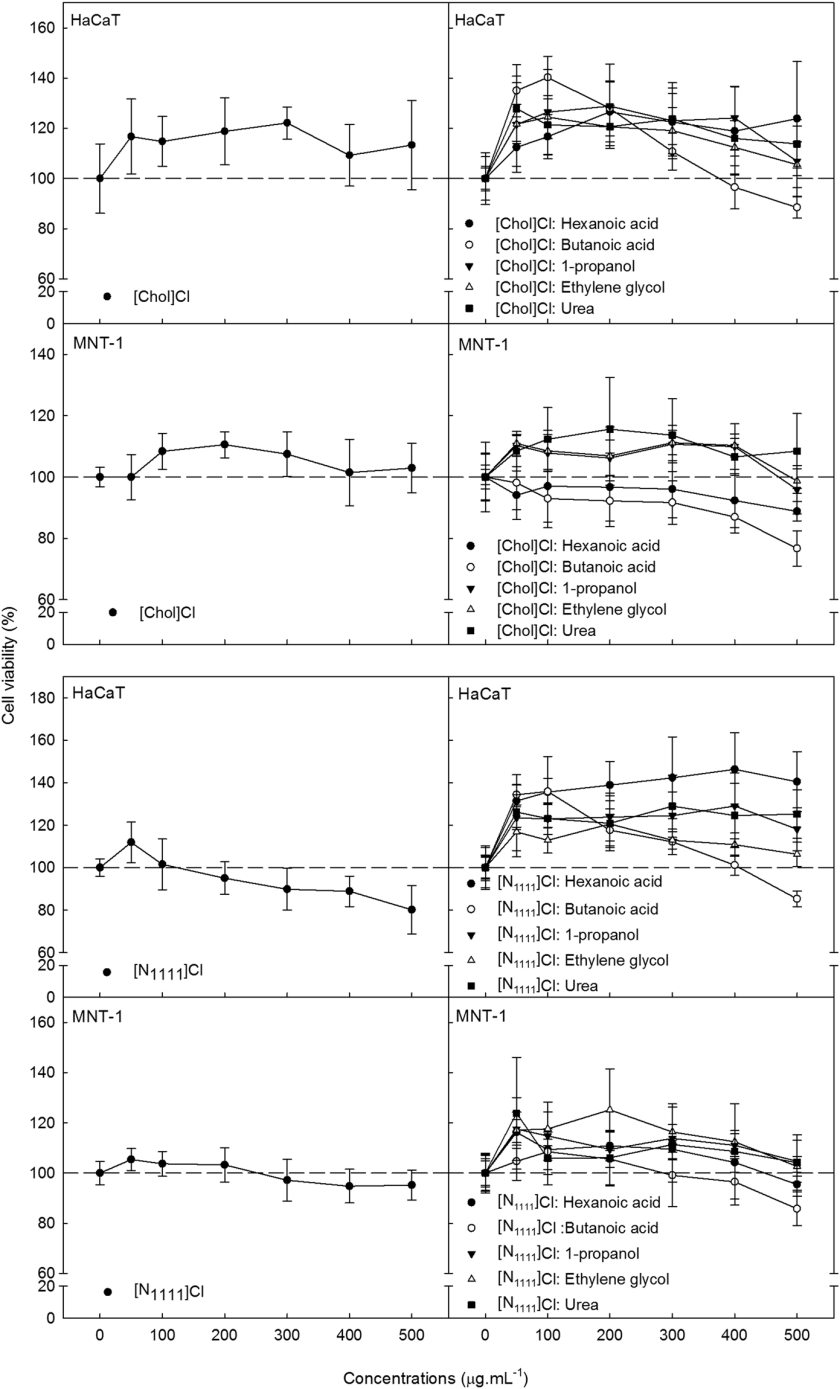
Cell viability of [Chol]Cl, and [N_1111_]Cl (as starting materials), and [Chol]Cl-based DES and [N_1111_]Cl-based DES for HaCaT and MNT-1 cells, after 72 h of exposure. Results are expressed as mean ± SD of three independent experiments.

[N_4444_]Cl in particular, produced interesting results in the present study. Both [N_4444_]Cl and all [N_4444_]Cl-based DES proved to be toxic for both skin cell lines tested. For components like [N_4444_]Cl, strangers to the cell or that are required in smaller amounts, the intercellular diffusion is restricted and they are retained in the cell membrane, having a more pronounced deleterious effect^29^. Actually, the accumulation of ammonium cations (above a specific threshold concentration) on cellular membranes can disrupt the lipid bilayer and induce cell death^45^. The toxic profile of [N_4444_]Cl-based DES was almost the same as that observed for [N_4444_]Cl (see Fig. 1). This translated in the yielding of a toxic DES regardless the HBD combined with [N_4444_]Cl, supporting the assumption that this HBA is the driver of the toxicity within any of the DES. The role as a toxicity driver was not as clear for the other HBA studied ([N_1111_]Cl and [Chol]Cl) (see Fig. 2). De Morais (2015)^21^ assessed the ecotoxicity of [CholCl-based DES towards the marine bacteria *A. fischeri* and found that the HBD (acids) had a preponderant effect in the toxicity. In the present study, although butanoic acid was found toxic to both cell lines, when used as an HBD, the resulting DES were not always cytotoxic and often increased cell viability. According to literature, butanoic acid and similar compounds can induce apoptosis in different types of cancer cells^46–49^, but it can also serve as an anti-inflammatory agent and as a source of energy in some non-tumoral cells^50^. These converse mechanisms of toxicity may concur to explain the inconsistent toxicity trends observed, which importantly question the role of HBD as toxicity drivers. Contrasting to our results but in agreement with those by De Morais *et al*.^21^, which showed the importance of HBD, Hayyan *et al*.^23^ also evidenced that HBD such as ethylene glycol, triethylene glycol, glycerine and urea can play a significant role in DES toxicity. Overall, it seems clear that DES toxicity is hardly generalizable and dependent on the actual HBA:HBD combination and ratio.

Contributing to the melting pot above collecting on inconsistent responses to DES exposure, hexanoic acid and 1-propanol produced different results when assessed with tumoral or non-tumoral cells. The 1-propanol increases the viability in the tumor cell line MNT-1, while hexanoic acid increased viability in non-tumoral cells, and induced toxicity in cancer cells. This suggests that their mechanism of action is different in tumor and non-tumoral cells. A similar compound, the 6-hydroxyhexanoic acid, showed that human fibroblasts (HGF-1) growth inhibition only occurs at a high exposure concentration of 20 mM^51^. The toxicity of hexanoic acid (*i.e*. caproic acid), as well as other related fatty acids (e.g. capric and caprylic acids) in cancer cells lines (colorectal, skin and mammary human cell lines) was already examined by Narayanan *et al*.^52^, who found cell viability significantly inhibited after 48 h of exposure by 75% to 90% compared to the control. These fatty acids are involved in the down-regulation of genes important to cell division and in the up-regulations of genes necessary to apoptosis in skin and colon cancer cells. Moreover, the relative activity of caspase-8 of cancer cells (human colorectal carcinoma cells - HCT-116) treated with these fatty acids was significantly higher than that found in control cells^52^. Briefly, caspase-8 is located at the top of the hierarchy of the caspase cascade leading to the apoptotic death of the cells^53^.

Also relevant was the increase viability in HaCaT caused by [Chol]Cl:urea and [N_1111_]Cl:urea, which did not happened in MNT-1 cells. [Chol]Cl serves as cellular raw material for the synthesis of phospholipids membranes such as phatidycholine and sphingomyelin^54^. Thus, intracellular choline availability is crucial for the metabolism both in normal and cancer cells^29^. Therefore, we were not expecting to find cytotoxic signals as [Chol]Cl or derived DES were tested. Although the use of [Chol]Cl is forbidden in cosmetic formulations in UE^55^, [Chol]Cl-based DES are widely used in many different applications^14,15,56–58^ and therefore it is important to study possible irritation symptoms due to dermal contact. Indeed, [Chol]Cl was not toxic for either cell lines and was able to increase cell viability, a pattern also generally depicted for [Chol]Cl-based DES, in particular for cells exposed to [Chol]Cl:butanoic acid. Urea is also a natural compound and it is used in many commercial skin care lotions and creams, due to its moisturizing properties^59^. Urea and urea-based DES could increase cell viability in HaCaT and MNT-1. These were expected results, but still they contrast with those by Hayyan *et al*.^23^ who found toxic effects induced by [Chol]Cl:urea and respective starting materials towards the skin cancer cells A375. In our study, only [N_4444_]Cl:ethylene glycol exhibited toxicity records (IC_50_ = 34.1 µg.mL^−1^) similar to those shown by Hayyan *et al*.^23^. Ethylene glycol belongs to a family of chemicals that exert their pharmacological and/or toxicological effect through biotransformation. In the case of ethylene glycol, biotransformation involves the conversion of a substance to its active metabolite causing the biological response^60^. Ethylene glycol has little intrinsic toxicological activity^61^, but its metabolite glycolic acid exhibits nonlinear kinetics and has a dose-dependent transition that can lead to the development of toxicity in animals under specific circumstances^60^. Our results may hence have been driven by glycolic acid rather than ethylene glycol.

Regarding the putative role of the HBD functional group, inconsistent trends could be highlighted from the dataset. For example, butanoic but not hexanoic acid was cytotoxic, and when conjugated with [N_1111_]Cl, both acids contributed to an increase in cell viability. Conversely, the use of acids as HBD such as oxalic acid^22^, malonic acid^30^, tartaric acid, and citric acid^28^, generally present increased toxicity. Even extrapolating the results obtained for acids as HBD towards other organisms^21^, the mechanisms are still not easy to understand. If the pH imposed by the presence of acids could play an important role, some of the results obtained in this work prove that this specific condition should not be used as an heuristic rule to explain the main mechanism of (cyto)toxicity, as the results of this work show. Moreover, none of the tested alcohols produced cytotoxic effects, but ethylene glycol increased cell viability when tested alone, as well as when tested as HBD with [Chol]Cl and [N_1111_]Cl, while 1-propanol elicited no effects in HaCaT, but increased MNT-1 viability when dosed singly and when used as HBD with [Chol]Cl and [N_1111_]Cl in both cell lines.

## Conclusions

DES have emerged in the last years as designer solvents with interesting properties and behavior, under the scope of “Green Chemistry”. Despite the increased attention given to DES, due to their promising applications in the cosmetic and pharmaceutical fields (just to mentioned a few), their characterization in terms of (cyto)toxicity is still very incomplete. In this context, most of the DES studied in this work were harmless for the cell lines HaCaT and MNT-1, even at high concentrations (500 μg.mL^−1^), thus both [Chol]Cl- and [N_1111_]Cl-based DES constitute promising benign compounds judging on their cytotoxic effects towards these two specific cell lines. Also, compounds like [Chol]Cl, ethylene glycol, hexanoic acid, urea, and all [Chol]Cl and [N_1111_]Cl-based DES allowed an increase in cell viability on the HaCaT cell line. Therefore, these compounds should be targets of future studies regarding their potential in skin regeneration. On the opposite, all the [N_4444_]Cl-based DES showed cytotoxicity. Moreover, the toxic profiles of these [N_4444_]Cl-based DES were similar to those exhibited by [N_4444_]Cl, suggesting that this HBA renders DES hazardous regardless the HBD used. Regarding the HBD, no consistent trends were obtained in the cytotoxic responses, with opposed effects being noticed between compounds bearing the same functional group. The increased cell viability caused by the majority of the tested compounds in non-tumoral cells (HaCaT) is an interesting aspect deserving further investigation. Still, it is important to understand the mechanisms behind this increase in cell viability, since it can represent cell proliferation promoting skin regeneration, but it can also indicate the possible activation of mutagenesis mechanisms.

## Supplementary information

Cytotoxicity profiling of deep eutectic solvents to human skin cells
