# Supplementary material for: Cytotoxicity profiling of deep eutectic solvents to human skin cells
Source: Sci Rep. 2019 Mar 8;9:3932. doi: 10.1038/s41598-019-39910-y (PMC6408470; doi:10.1038/s41598-019-39910-y)
Supplement: Supplementary file 1 — Cytotoxicity profiling of deep eutectic solvents to human skin cells [file 41598_2019_39910_MOESM1_ESM.pdf]

Supplementary Information

# Cytotoxicity profiling of deep eutectic solvents to human skin cells

Macário[1], I.P.E., Oliveira[1,2], H.*, Menezes[1], A.C., Ventura[2], S.P.M., Pereira[1], J.L., Gonçalves[1,3], A.M.M., Coutinho[2], J.A.P, Gonçalves[1], F.J.M.

1 - Department of Biology & CESAM, University of Aveiro, 3810-193 Aveiro, Portugal
2 - CICECO - Aveiro Institute of Materials & Department of Chemistry, University of Aveiro, 3810-193 Aveiro, Portugal
3- MARE, Department of Life Sciences, Faculty of Science and Technology, University of Coimbra, 3004-517 Coimbra, Portugal

* Corresponding author e-mail: holiveira@ua.pt

**Table S1**. ANOVA summary table for viability assays conducted for each HBA, HBD and DES. Test (*F*) statistics and degrees of freedom are shown. MS stands for the mean squares (= variance) of the error term in the ANOVA.

| **Chemical compound** | **Cell line** | **Source of variation** | **Degrees of freedom** | **Adjusted MS** | **Test statistic (F)** | ***P*** |
|---|---|---|---|---|---|---|
| 1-propanol | HaCaT | Treatment | 6 | 242.814 | 2.399 | < 0.05 |
| | | Error | 56 | 101.234 | | |
| | MNT-1 | Treatment | 6 | 606.469 | 7.800 | < 0.001 |
| | | Error | 56 | 77.752 | | |
| [Chol]Cl | HaCaT | Treatment | 6 | 473.349 | 2.754 | < 0.05 |
| | | Error | 56 | 171.892 | | |
| [$N_{1111}$]Cl | HaCaT | Treatment | 6 | 957.494 | 11.330 | < 0.001 |
| | | Error | 56 | 84.506 | | |
| | MNT-1 | Treatment | 6 | 167.694 | 4.484 | < 0.001 |
| | | Error | 56 | 37.399 | | |
| [$N_{4444}$]Cl | MNT-1 | Treatment | 6 | 4165.981 | 87.661 | < 0.001 |
| | | Error | 56 | 47.524 | | |
| Hexanoic acid | HaCaT | Treatment | 6 | 836.197 | 6.570 | < 0.001 |
| | | Error | 56 | 127.267 | | |
| | MNT-1 | Treatment | 6 | 1172.941 | 54.774 | < 0.001 |
| | | Error | 56 | 21.414 | | |
| Butanoic acid | MNT-1 | Treatment | 6 | 11659.994 | 508.086 | < 0.001 |
| | | Error | 56 | 22.949 | | |
| Urea | HaCaT | Treatment | 6 | 303.124 | 4.158 | < 0.05 |
| | | Error | 56 | 72.897 | | |
| | MNT-1 | Treatment | 6 | 383.174 | 4.904 | < 0.001 |
| | | Error | 56 | 78.138 | | |
| [Chol]Cl: Hexanoic acid | MNT-1 | Treatment | 6 | 118.368 | 1.330 | 0.259 |
| | | Error | 56 | 88.991 | | |
| [Chol]Cl: Butanoic acid | HaCaT | Treatment | 6 | 3734.246 | 56.332 | < 0.001 |
| | | Error | 56 | 66.290 | | |
| | MNT-1 | Treatment | 6 | 537.069 | 9.339 | < 0.001 |
| | | Error | 56 | 57.510 | | |
| [Chol]Cl: Urea | HaCaT | Treatment | 6 | 739.986 | 7.583 | < 0.001 |
| | | Error | 56 | 97.579 | | |
| [Chol]Cl: 1-propanol | HaCaT | Treatment | 6 | 1060.256 | 4.818 | < 0.001 |
| | | Error | 56 | 220.060 | | |
| | MNT-1 | Treatment | 6 | 295.371 | 8.254 | < 0.001 |
| | | Error | 56 | 35.787 | | |
| [Chol]Cl: Ethylene glycol | HaCaT | Treatment | 6 | 757.471 | 10.479 | < 0.001 |
| | | Error | 56 | 72.282 | | |
| [$N_{1111}$]Cl: Hexanoic acid | HaCaT | Treatment | 6 | 2174.395 | | < 0.001 |
| | | Error | 56 | 206.575 | | |
| | MNT-1 | Treatment | 6 | 448.110 | 17.329 | < 0.001 |
| | | Error | 56 | 25.860 | | |

| | | | | | | |
|---|---|---|---|---|---|---|
| [$N_{1111}$]Cl: Butanoic acid | HaCaT | Treatment | 6 | 3109.968 | 99.597 | < 0.001 |
| | | Error | 56 | 31.226 | | |
| | MNT-1 | Treatment | 6 | 513.288 | 5.971 | < 0.001 |
| | | Error | 56 | 85.966 | | |
| [$N_{1111}$]Cl: Urea | HaCaT | Treatment | 6 | 847.578 | 6.185 | < 0.001 |
| | | Error | 56 | 137.030 | | |
| | MNT-1 | Treatment | 6 | 518.435 | 2.445 | < 0.05 |
| | | Error | 56 | 212.003 | | |
| [$N_{1111}$]Cl: Ethylene glycol | HaCaT | Treatment | 6 | 412.118 | 5.986 | < 0.001 |
| | | Error | 56 | 68.846 | | |
| [$N_{4444}$]Cl: Hexanoic acid | MNT-1 | Treatment | 6 | 3817.235 | 84.844 | < 0.001 |
| | | Error | 56 | 44.991 | | |
| [$N_{4444}$]Cl: Butanoic acid | MNT-1 | Treatment | 6 | 3364.697 | 199.669 | < 0.001 |
| | | Error | 56 | 16.851 | | |
| $N_{4444}$]Cl: 1-propanol | MNT-1 | Treatment | 6 | 7321.778 | 463.362 | < 0.001 |
| | | Error | 56 | 15.801 | | |
| $N_{4444}$]Cl: Urea | HaCaT | Treatment | 5 | 10521.082 | 585.114 | < 0.001 |
| | | Error | 48 | 17.981 | | |

**Table S2.** One-way ANOVA on ranks summary table for viability assays conducted for each HBA, HBD and DES. Test (*H*) statistics and degrees of freedom are shown.

| **Chemical compound** | **Cell line** | **Degrees of freedom** | **Test statistics (H)** | ***P*** |
|---|---|---|---|---|
| Ethylene glycol | HaCaT | 6 | 36.060 | < 0.001 |
| | MNT-1 | 6 | 32.135 | < 0.001 |
| [Chol]Cl | MNT-1 | 6 | 17.750 | < 0.05 |
| [$N_{4444}$]Cl | HaCaT | 6 | 57.909 | < 0.001 |
| Butanoic acid | HaCaT | 6 | 56.876 | < 0.001 |
| [Chol]Cl: Hexanoic acid | HaCaT | 6 | 21.917 | < 0.05 |
| [Chol]Cl: Urea | MNT-1 | 6 | 12.567 | 0.05 |
| [Chol]Cl: Ethylene glycol | MNT-1 | 6 | 31.128 | < 0.001 |
| [$N_{1111}$]Cl: 1-propanol | HaCaT | 6 | 22.018 | < 0.05 |
| | MNT-1 | 6 | 25.649 | < 0.001 |
| [$N_{1111}$]Cl: Ethylene glycol | MNT-1 | 6 | 36.875 | < 0.001 |
| [$N_{4444}$]Cl: Hexanoic acid | HaCaT | 6 | 57.981 | < 0.001 |
| [$N_{4444}$]Cl: Butanoic acid | HaCaT | 6 | 60.187 | < 0.001 |
| [$N_{4444}$]Cl: 1-propanol | HaCaT | 6 | 52.086 | < 0.001 |
| [$N_{4444}$]Cl: Ethylene glycol | HaCaT | 6 | 48.516 | < 0.001 |
| [$N_{4444}$]Cl: Urea | MNT-1 | 6 | 50.356 | < 0.001 |

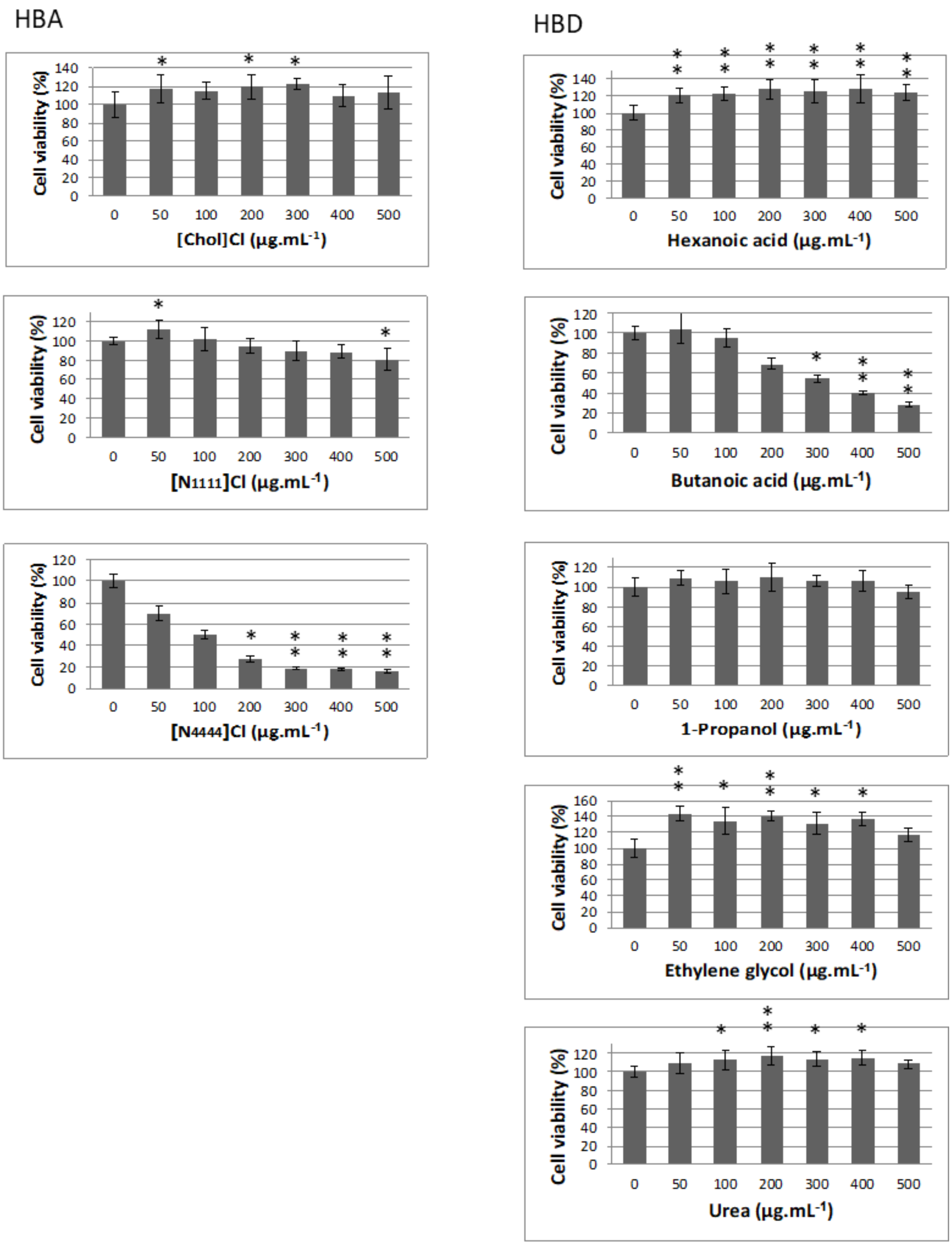


**Figure S1:** Cell viability of HaCaT cell line after 72h of exposure to starting materials. Results are expressed as mean ± SD of three independent experiments. Statistical analysis: $^*p < 0.05$ vs control; $^{**}p < 0.001$ vs control

HBA:HBD 1:1

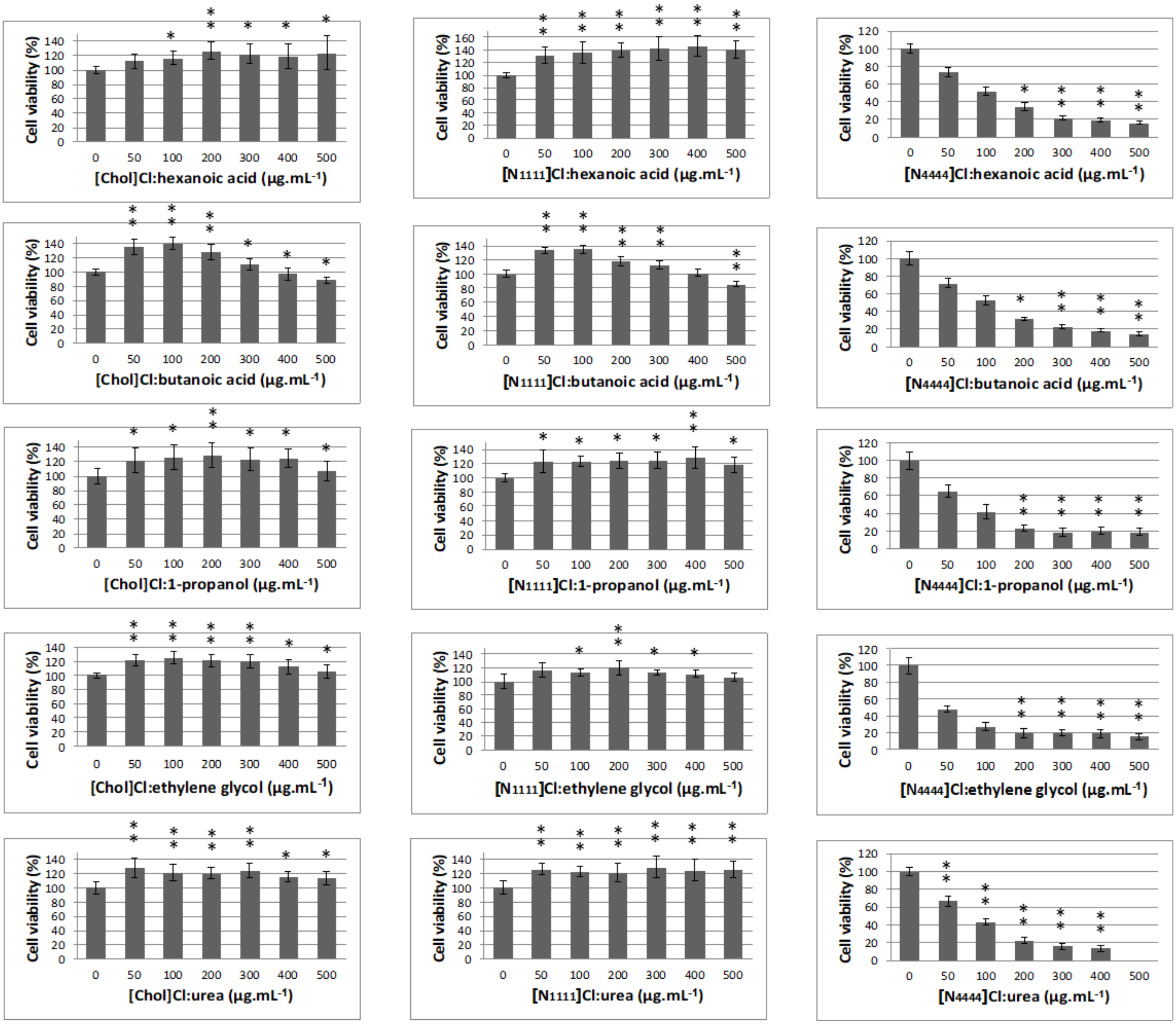


**Figure S2:** Cell viability of HaCaT cell line after 72h of exposure to DES. Results are expressed as mean ± SD of three independent experiments. Statistical analysis: *$p < 0.05$ vs control; **$p < 0.001$ vs control

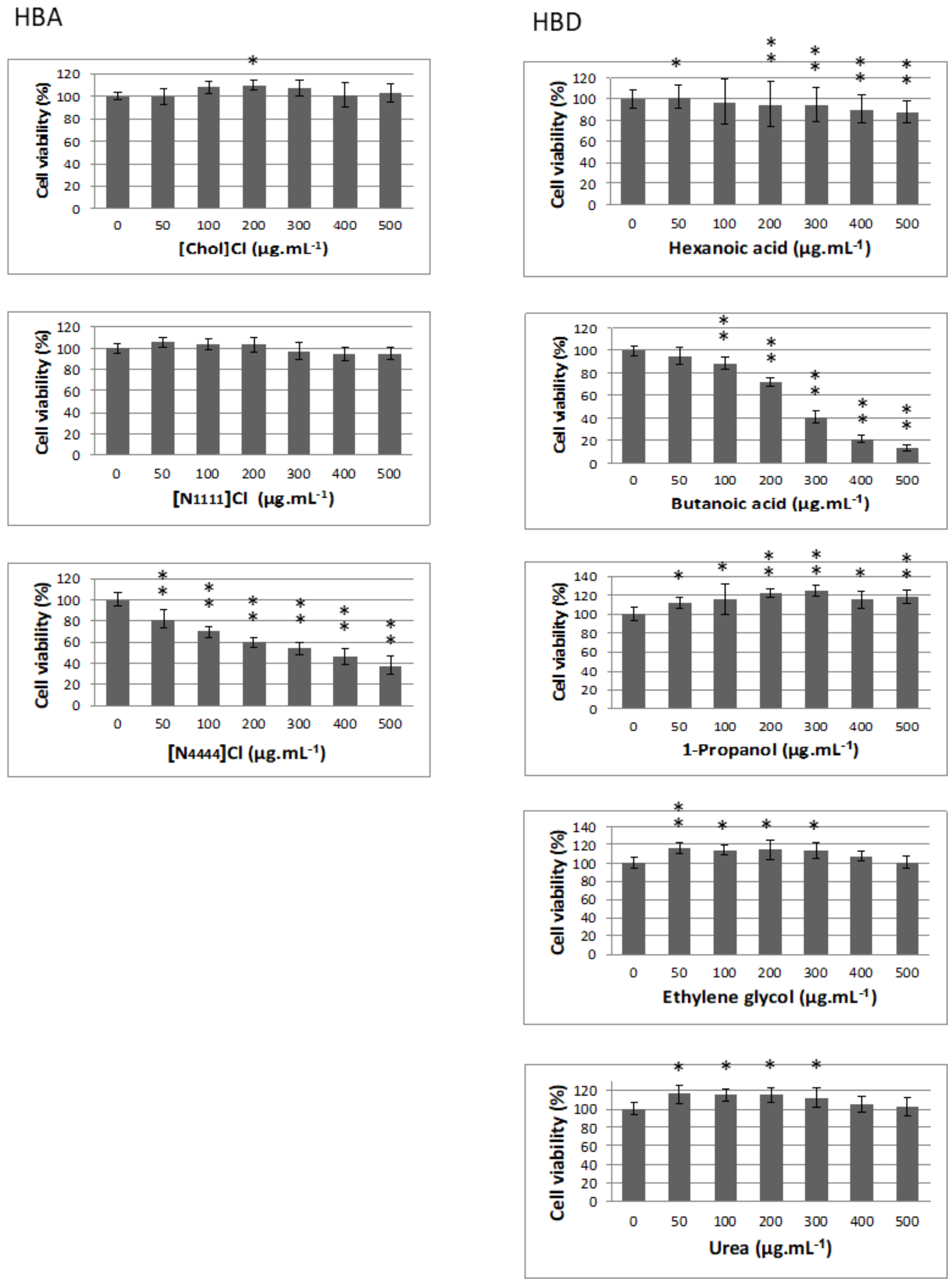


**Figure S3:** Cell viability of MNT-1 cell line after 72h of exposure to starting materials. Results are expressed as mean ± SD of three independent experiments. Statistical analysis: *$p < 0.05$ vs control; **$p < 0.001$ vs control

HBA:HBD 1:1

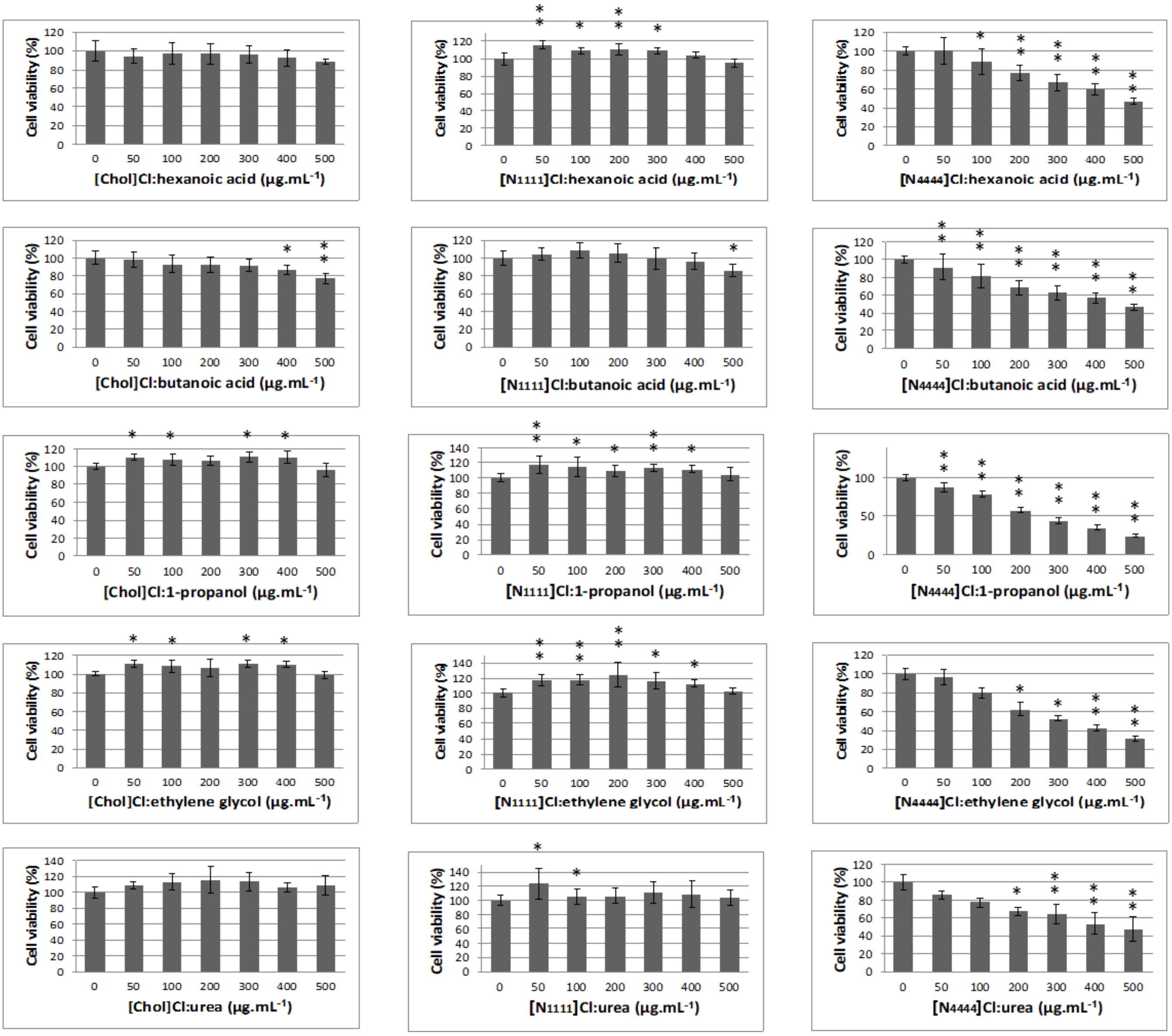


**Figure S4:** Cell viability of MNT-1 cell line after 72h of exposure to DES. Results are expressed as mean ± SD of three independent experiments. Statistical analysis: *$p < 0.05$ vs control; **$p < 0.001$ vs control
